# Surgical treatment of valve endocarditis in high-risk patients and predictors of long-term outcomes

**DOI:** 10.1038/s41598-021-03602-3

**Published:** 2021-12-20

**Authors:** Giuseppe Nasso, Giuseppe Santarpino, Marco Moscarelli, Ignazio Condello, Angelo Maria Dell’Aquila, Armin Darius Peivandi, Mario Gaudino, Flavio Fiore, Pasquale Mastroroberto, Nicola Di Bari, Giuseppe Speziale

**Affiliations:** 1 Department of Cardiac Surgery, Anthea Hospital, GVM Care and Research, Via Camillo Rosalba, 35/38 Bari, Italy; 2 Department of Cardiac Surgery, Paracelsus Medical University, Nuremberg, Germany; 3 Department of Clinical and Experimental Medicine, Magna Graecia University, Catanzaro, Italy; 4 Department of Cardiac Surgery, University of Münster, Munster, Germany; 5 Department of Cardiothoracic Surgery, Weill Cornell Medicine, New York, NY USA

**Keywords:** Diseases, Health care, Medical research, Pathogenesis

## Abstract

Infective endocarditis represents a surgical challenge associated with perioperative mortality. The aim of this study is to evaluate the predictors of operative mortality and long-term outcomes in high-risk patients. We retrospectively analyzed 123 patients operated on for infective endocarditis from January 2011 to December 2020. Logistic regression model was used to identify prognostic factors of in-hospital mortality. Long term follow-up was made to asses late prognosis. Preoperative renal failure, an elevation EuroSCORE II and prior aortic valve re-replacement were found to be preoperative risk factors significantly associated with mortality. In-hospital mortality was 27% in patients who had previously undergone aortic valve replacement (*n* = 4 out of 15 operated, *p* = 0.01). Patients who were operated on during the active phase of infective endocarditis showed a higher mortality rate than those operated on after the acute phase (16% vs. 0%; *p* = 0.02). The type of prosthesis used (biological or mechanical) was not associated with mortality, whereas cross-clamp time significantly correlated with mortality (mean cross-clamp time 135 ± 65 min in dead patients vs. 76 ± 32 min in surviving patients; *p* = 0.0005). Mean follow up was 57.94 ± 30.9 months. Twelve patients died (11.65%). Among the twelve mortalities, five were adjudicated to cardiac causes and seven were non-cardiac (two cancers, one traumatic accident, one cerebral hemorrhage, two bronchopneumonia, one peritonitis). Overall survival probability (freedom from death, all causes) at 3, 5, 7 and 8 years was 98.9% (95% CI 97–100%), 96% (95% CI 92–100%), 85.9% (95% CI 76–97%), and 74% (95% CI 60–91%) respectively. Our study demonstrates that an early surgical approach may represent a valuable treatment option for high-risk patients with infective endocarditis, also in case of prosthetic valve endocarditis. Although several risk factors are associated with higher mortality, no patient subset is inoperable. These findings can be helpful to inform decision-making in heart team discussion.

## Introduction

Infective endocarditis (IE), particularly on a heart valve prosthesis, represents a surgical challenge mostly for two reasons. First, prosthetic valve endocarditis (PVE) and native valve endocarditis (NVE) are a life-threating diseases^1,2^. Second, patients with IE are increasingly of advanced age and at high risk. In addition, many patients considered at intermediate-to-high risk or inoperable that have undergone transcatheter aortic valve replacement are also susceptible to infective endocarditis on these prostheses^1,2^.

There are well-known patient subsets that are at higher risk of mortality if undergoing cardiac surgery, due to anatomical features of valve heart disease and coexistent comorbidities^3^, with more than 10% of patients considered to be at too high risk for surgery^1^. Although surgical techniques, prosthetic models and anesthetic management have constantly improved over the last years^3^, the increasing number of patients at higher risk for surgery may affect operative success, particularly in terms of higher mortality.

The aim of this study is to evaluate predictors of operative mortality and mid-long term outcomes in high-risk patients with endocarditis.

## Materials and methods

### Patients

**Figure 1:**
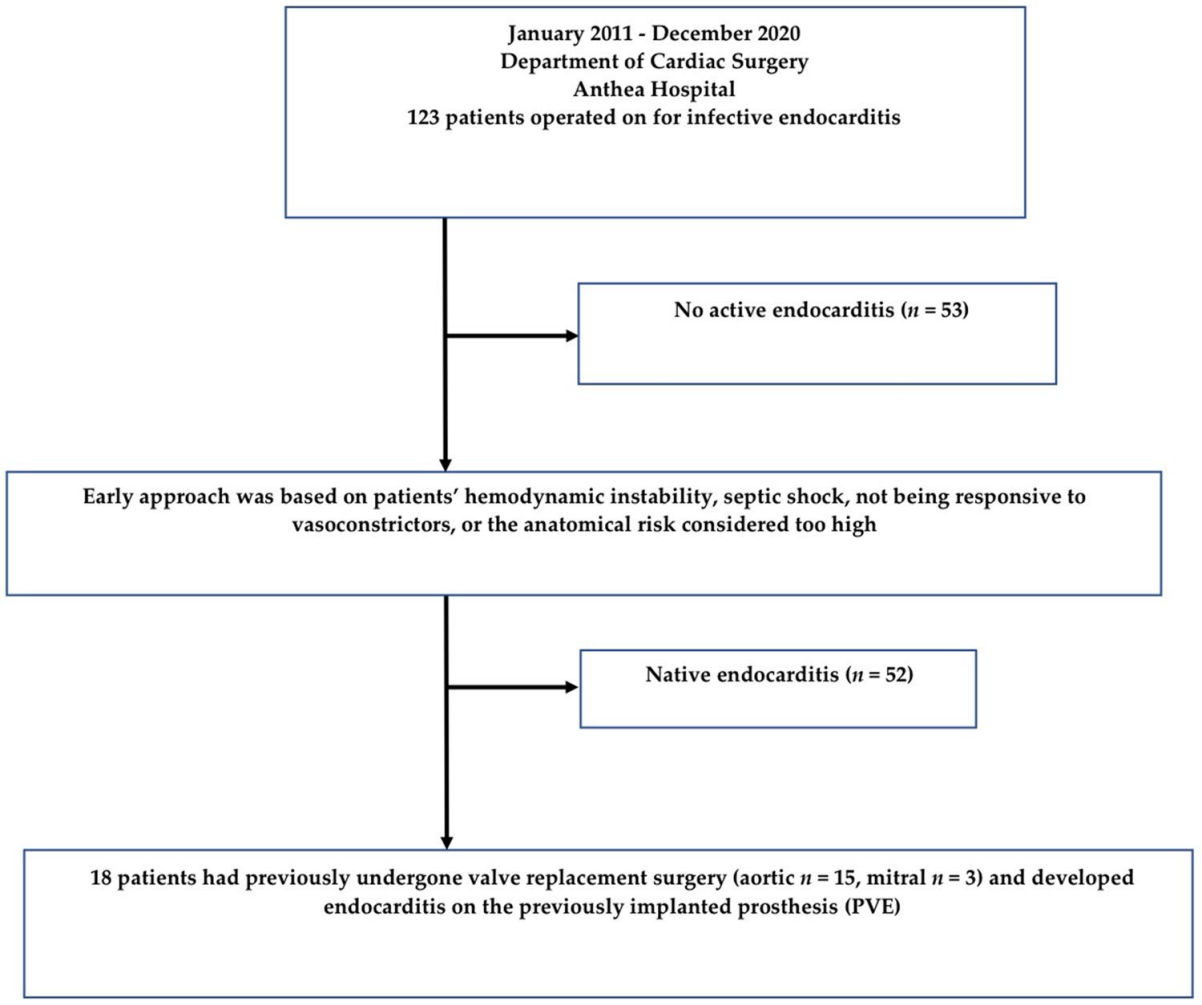
Flow-chart showing the characteristics of the study population.

We retrospectively analyzed all patients operated on for acute IE unresponsive to antibiotic therapy from January 2011 to December 2020 at our Department of Cardiac Surgery, Anthea Hospital GVM Care & Research, Bari, Italy. Complete data collection was available for 123 patients. Of these, 73 patients (59.35%) were men and 50 (40.65%) women, 8 (6.5%) patients developed native tricuspid valve endocarditis, 89 (72.36%) native aortic valve endocarditis, and 51 (41.46%) native mitral valve endocarditis. Eighteen (14.63%) patients had previously undergone valve replacement surgery (mitral or aortic) and developed endocarditis on the previously implanted prosthesis. Of these, at first operation, 15 (12.2%) underwent aortic valve replacement (AVR) and 3 (2.44%) mitral valve replacement (MVR). Fifty-four (43.9%) patients received a biological valve prosthesis and 69 (56.1%) mechanical valve prosthesis. Fifty-eight (47.15%) patients had active endocarditis and 53 (43.09%) no active endocarditis (Fig. 1; Tables 1, 2).

**Table 1:** Preoperative characteristics.

Variables	Total	%/±	Dead	% Dead	Alive	% Alive	*p*-value univariate Log regression
Age, years	63.78	13.46	68.8	9.8	63.34	13.68	0.2267
Male sex	73	59.35	4	5%	69	95%	0.2
Female sex	50	40.65	6	12%	44	88%	0.2
Tricuspid valve disease	8	6.5	1	13%	7	88%	0.64
Aortic valve disease	89	72.36	9	10%	80	90%	0.22
Mitral valve disease	51	41.46	6	12%	45	88%	0.22
Diabetes	18	14.63	2	11%	16	89%	0.62
NIDDM	14	11.38	2	14%	12	86%	0.38
Diabetes diet	3	2.44	0	0%	3	100%	1
IDDM	1	0.81	0	0%	1	100%	1
Hypercholesterolemia	62	50.41	5	8%	57	92%	0.98
Hypertension	99	80.49	7	7%	92	93%	0.39
Ex smoker	19	15.45	2	11%	17	89%	0.68
Current smoker	17	13.82	1	6%	16	94%	0.72
Gastrointestinal disease	10	8.13	0	0%	10	100%	1
Renal dysfunction	10	8.13	3	30%	7	70%	0.02
Dialysis	2	1.63	0	0%	2	100%	1
Respiratory disease	10	8.13	0	0%	10	100%	1
Cerebro-vascular disease	7	5.69	0	0%	7	100%	1
Liver disease	53	43.09	0	0%	53	100%	1
Cancer	2	1.63	1	50%	1	50%	0.08
Neurologic dysfunction	3	2.44	1	33%	2	67%	0.15
Peripheral artery disease	2	1.63	0	0%	2	100%	1
Atrial fibrillation/flutter	23	18.7	2	9%	21	91%	0.91
Pacemaker	3	2.44	1	33%	2	67%	0.15
REDO (re-AVR)	15	12.2	4	27%	11	73%	0.01
REDO (re-MVR)	3	2.44	0	0%	3	100%	1
EuroSCORE	8.86	4.23	14.1	3.35	8.4	3.99	0.0006
Log EuroSCORE	0.18	0.2	0.45	0.23	0.15	0.17	0,0049
EuroSCORE II	14.42	19.09	47.39	22.91	11.08	15.21	0.0007

Values are presented as the mean ± SD or n (%). There were no missing data.

*NIDDM* non insuline dependent diabetes mellitus; *IDDM* insuline dependent diabetes mellitus; *AVR* aortic valve replacement; *MVR* mitral valve replacement;* EuroSCORE* European System for cardiac Operative Risk Evaluation.

**Table 2:** Operative characteristics.

Variables	Total	%/ ±	Dead	% Dead	Alive	% Alive	*p*-value univariate Log regression
Active endocarditis	58	47.15	9	16%	49	84%	0.02
No active endocarditis	53	43.09	0	0%	53	100%	1
Aortic cross-clamp time	81.25	39.4	135.6	65.18	76.05	32.01	0.0005
Biological prosthesis	54	43.9	7	13%	47	87%	0.1
Mechanical prosthesis	69	56.1	3	4%	66	96%	0.1

Values are presented as the mean ± SD or n (%). There were no missing data.

The diagnosis of IE was based on the results of echocardiography and blood cultures, and made according to the Duke criteria^4^. Active endocarditis is defined as patients are still under antibiotics at the time of surgery. Surgical timing is classified into “Emergent” and “Urgent”. Emergent surgery refers to an operation that begins within 24 h after the diagnosis of IE or heart failure is made while urgent surgery when the patients are not electively admitted for the surgery but they require surgery during hospitalization according to the ESC and AHA/ACC recommendations^4^. We considered only patients undergoing surgery within 7 days of hospitalization. The decision of an emergent or urgent approach was based on patients’ hemodynamic instability, septic shock, not being responsive to vasoconstrictors, or the anatomical risk considered too high (e.g. detachment of the valve prosthesis with annular abscess and severe perivalvular leakage).

The decision for an emergent or urgent approach has always been shared within a team including a cardiac surgeon, a cardiologist and an anesthesiologist, in consultation with other professionals (infectious disease specialist, neurologist in case of concomitant brain injury, general surgeon in case of embolic damage to the abdominal organs, vascular surgeon in case of peripheral embolism with limb ischemia to be evaluated concomitantly).

All patients were contacted for follow-up every year after the operation and checked with regular clinical and echocardiographic visits.

The GVM Care & Research review board approved the study and informed consent was obtained from all subjects involved in the study. All methods were performed in accordance with the relevant guidelines and regulations by including a statement.

### Surgery

Pre-operative echocardiography transesophageal (TEE) was routinely used. All interventions were performed under general anesthesia and complete median sternotomy or re-sternotomy. By institutional protocol, aortic and bicaval cannulation for extracorporeal circulation were performed prior to sternotomy while femoral vein and arteria cannulation prior to re-sternotomy. After external aortic cross-clamping, cold (4 °C) intermittent antegrade blood cardioplegia into the coronary ostia was used for myocardial protection. The valve and surrounding infected tissue were carefully inspected. The native valve or prosthetic valve was entirely removed and sent to the laboratory for culture examination. If the infection involves the annulus and surrounding tissue, all infected material, foreign bodies, and necrotic tissues were removed to minimize the residual infectious burden and provide optimal access for host defense and antimicrobial therapy.

The aortic or mitral annulus and the mitro-aortic continuity were cleaned, the abscesses were emptied and washed with disinfectant solution.

For native aortic valve, when a localized abscess is not larger than a single aortic cusp, the aortic annuls was reconstructed by plicating the defect between pledgeted mattress sutures placed just below the native aortic annulus and the sewing ring of the prosthetic stented valve during AVR. In the presence of abscess cavities or tissue defects larger than one aortic cusp without aorto-ventricular dehiscence, the defect on the aortic annulus was reconstructed with use of an autologous pericardium, bovine pericardium, and other materials, and pledgeted sutures placed on this patch during AVR. For patients with prosthetic aortic endocarditis, in the presence of abscess involving the aortic annulus, the new prosthetic valve was positioned inside the aortic root, exactly at the level of the sinuses of Valsalva, far from the destroyed annulus and without use of patch for reconstruction of mitro-aortic curtain. In these cases the abscess was left open to drain to allow a continuous drainage of the cavity and an easier achievement by antibiotics. Special attention was paid to the patency of the coronary ostia in the vicinity of the new prosthesis.

In patient with mitral valve endocarditis after deep debridement of infected tissue the standard technique for replacement was used. When annulus reconstructed is needed, autologous pericardium, bovine pericardium, and other materials were used.

The choice of the prosthetic model to be implanted (mechanical, biological, stentless, sutureless) was based on the patient’s clinical and anatomical conditions, life expectancy and, if possible, will.

All patients with tricuspid valve endocarditis underwent tricuspid valve replacement with biological prosthesis.

### Registered variables and statistical analysis

Preoperative, perioperative, and postoperative variables were retrospectively recorded in an institutional database. The assumption of normality of each variable distribution was tested with the Shapiro–Wilk test. Normally distributed variables were reported as the mean ± standard deviation or median. Categorical variables are reported as number and percentage. An analysis for the identification of preoperative risk factors associated with postoperative mortality was carried out. All preoperative variables available in our database were entered into a logistic univariate analysis in order to highlight significant differences between survivors and deaths. In the second step, all variable with a *p* value under 0.2 were entered into the multivariable analysis with stepwise selection. Moreover to avoid collinearity, variables expressing or including similar parameters were intentionally omitted for the construction of the multivariable model. The overall survival probability was analyzed using the Kaplan–Meier method and corresponding survival curves. All analyses were performed using R 2.13.2 software (R Development Core Team, Vienna, Austria). The threshold for statistical significance was *p* < 0.05.

### Ethics approval and consent to participate

The study was evaluated and approved by the institutional board for clinical trials, Anthea Hospital GVM Care & Research (internal protocol; decision 2021 Feb) and Informed consent was obtained from all subjects involved in the study.

### Consent for publication

All authors have read and agreed to the published version of the manuscript.

## Results

The preoperative characteristics of the study patients are reported in Table 1. Among preoperative risk factors, preoperative renal failure, EuroSCORE II and prior aortic valve replacement were found to be significantly associated with mortality. In-hospital mortality was 16.26% (20 patients) in the study patients, and 27% in patients who had previously undergone aortic valve replacement (*n* = 4 out of 15 operated, *p* = 0.01).

Patients who were operated on during the active phase of infective endocarditis showed a higher mortality rate than those operated on after the acute phase (16% vs. 0%; *p* = 0.02). The type of prosthesis used (biological or mechanical) was not associated with mortality, whereas cross-clamp time significantly correlated with mortality (mean cross-clamp time 135 ± 65 min in dead patients vs. 76 ± 32 min in surviving patients; *p* = 0.0005) (Table 2).

After adjusting for confounders, EuroSCORE II (*p* = 0.022, odds ratio [OR] 1.047, 95% confidence interval [CI] 1.007–1.090) (i nuovi numeri Euroscore II *p* = 0.0037 coeficient (0.059) odd ratio 1.061, 95% CI 1.019–1.1052 and for Xclamp 0 = 0.0889) and cross clamping time (*p* < 0.001, (coefficient 0.017) OR 1.031, 95% CI 1.012–1.049) were found to be independent predictors of 30-day mortality.

### Follow-up

All patients were followed up at our institution every 6 months after the procedure. Visits included a physical examination, 12-lead electrocardiography, trans-thoracic echocardiography. A TEE was performed once a year. Mean follow-up was 57.94 ± 30.9 months (median 59 months, interquartile range 32.5–86). There was no lost at follow-up.

Twelve patients died (11.65%). Among the twelve mortalities, five were adjudicated to cardiac causes and seven were non-cardiac (two cancers, one traumatic accident, one cerebral hemorrhage, two bronchopneumonia, one peritonitis) (Tables 3, 4).

**Table 3:** Postoperative characteristics.

Outcomes (N = *123*)	Values
Respiratory failure or lung complications^a^	9 (7.3%)
Ischemic stroke	8 (6.5%)
Coma	5 (4%)
Inotropic support^b^	30 (24.4%)
Cardiogenic shock	10 (8.1%)
Sternal wound infection	2 (1.6%)
Septic shock	4 (3.25%)
Dialysis	9 (7.3%)
CRF	18 (14.6%)
De novo pacemaker	4 (3.25%)
De novo AF	14 (11.38%)
Re-opening for bleeding	7 (5.7%)
ICU stay (days)	8 ± 7.2
30-day mortality	20 (16.26%)

Values are presented as the mean ± SD or n (%). There were no missing data.

*CRF* chronic renal failure; *AF* atrial fibrillation; *ICU* intensive care unit.

^a^Respiratory failure includes prolonged mechanical ventilation time (> 48 h), need for reintubation, and pneumonia; lung complications include persistent airspace or pneumothorax and significant pleural effusion; ^b^Defined as the use of adrenaline and/or dopamine and/or dobutamine and/or phosphodiesterase inhibitor/levosimendan.

**Table 4:** Outcomes at follow-up.

Outcomes (N = 103)	Values
Permanent pacemaker implantation, n (%)	22 (21.4)
Stroke, TIA, n (%)	15 (14.6)
Peri-prosthetic leak, n (%)	
Grade 1 or trivial	59 (57.3)
Grade 2	0
Grade 3	0
Aortic gradient, mmHg, mean ± SD	14 ± 9.3
Pulmonary pressure, mmHg, mean ± SD	43.3 ± 8.3
LVEF %, mean ± SD	42 ± 9

Data are presented as mean ± SD, or number and frequency (%).

*LVEF* left ventricle ejection fraction, *TIA* transient ischemic attack.

Overall survival probability (freedom from death, all causes) at 3, 5, 7 and 8 years was 99% (95% CI 97–100%), 96% (95% CI 92–100%), 86% (95% CI 76–97%), and 74% (95% CI 60–91%) respectively.

**Figure 2:**
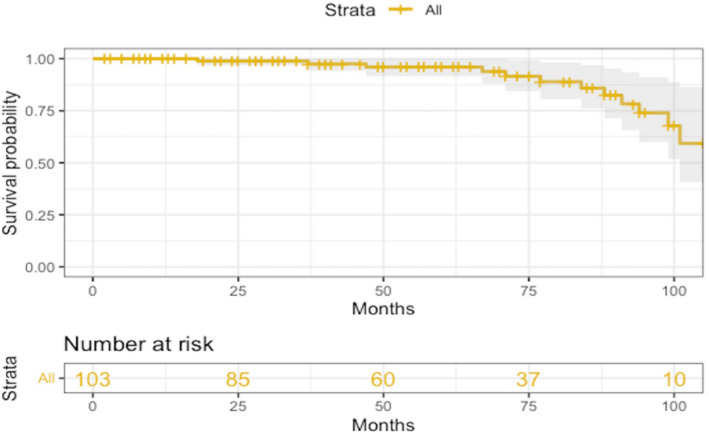
Kaplan–Meier curves: overall survival probability (freedom from death, all causes) at 3, 5, 7 and 8 years was 99% (95% CI 97–100%), 96% (95% CI 92–100%), 86% (95% CI 76–97%), and 74% (95% CI 60–91%) respectively.

At follow-up no patients presented with moderate or severe (grade 2–3) prosthesis/aortic or mitral regurgitation; mild to trivial aortic or mitral regurgitation was found in 59 patients (57.3%). Mean trans-prosthesis aortic gradient was 14 ± 9.3 mmHg; one patient underwent TAVI for prosthesis structural deterioration (stenosis). Among survivors, 90 and 13 were in NYHA class 2 and 3 respectively; transient or permanent ischemic accidents were observed in 12 patients. No patients had recurrence endocarditis (Fig. 2).

## Discussion

Our study described the experience of our center with the surgical treatment of patients with endocarditis. Our discussion will be focused on the results obtained in high-risk patients with endocarditis undergoing early surgery (within 7 days of admission), often considered inoperable due to their comorbidities and clinical status. That gives originality to our study; to operate high risk patients up to compassionate indications.

In a matched retrospective cohort study of 139 dialysis patients, Farrington et al. reported that the risk of PVE and death after valve replacement was significantly higher in dialysis patients than in patients without dialysis^5^. However, the mortality rate was less than 20% and the timing of intervention was unknown. This finding is consistent with our results, suggesting that operation should be taken into consideration also in patients at higher risk, even if burdened by a high mortality rate. Likewise, we also recorded a correlation between preoperative renal insufficiency and mortality. In this population, any delay to intervention or medical treatment alone can result in higher mortality^6^. Although we did not include a control group on medical therapy, the mortality rate we recorded in operated patients was lower than in previous studies with a control group^6^. Additionally, it is well-known that endocarditis is associated with high mortality particularly when urgent surgery is needed^7^. In the analysis by Revilla et al.^7^, the main indication for urgent surgery was heart failure. This could also be ascribed to the waiting time until surgery leading to worsening of heart failure and hemodynamic instability in emergency. In our opinion, a “wait-and-see” approach may have resulted in critical clinical conditions and extensive anatomical injury^8,9^.

Surgical technique also plays a role, especially if a complex procedure for endocarditis with annular or root destruction should be performed, though associated with higher mortality^10^. In case of aortic root involvement, several prosthetic models seem helpful in facilitating the radicality of the procedure or favoring resistance to recurrence^11–13^. In our population of high-risk patients, we chose in some patients to adopt the simplest technique by minimizing ischemic time, and priority was given to the removal of the infected tissue and implantation of the new prosthesis in an area distant from the previous one. In other words, radicality is key but a fast procedure is very important because prolonged cross-clamp time is correlated with postoperative mortality. Obviously, out study doesn´t have the aim to create a risk score using the cross-clamp time; it cannot be assessed preoperatively.

Age is another factor to be taken into consideration when evaluating operability. In patients undergoing surgery for infective endocarditis, regardless of whether native valve endocarditis or PVE, available evidence shows that advanced age is associated with higher mortality rates up to 20% in patients above 75 years^14^. Of the 4 patients aged > 75 years included in our PVE population, only one died, supporting surgical indication also in this high-risk patient subset undergoing re-operation. Advanced age is a risk factor common to all interventions in cardiac surgery and not only for endocarditis per se.

We believe there may be a “bias” towards some patients who are considered to be at too high risk for surgery. For instance, patients undergoing transcatheter aortic valve implantation (TAVI) have been found to have a risk for developing infective endocarditis similar to those undergoing surgical aortic valve replacement, and no differences have been reported between these two patient subsets when undergoing surgery/re-surgery^15–17^. These findings should prompt us to evaluate operability and the risk of mortality at the time of first intervention. Patients undergoing TAVI and re-operated for endocarditis, given the historical period and the chronological sequence, are at least at intermediate risk if not considered inoperable^2^. It is also likely that, in some specific conditions related to endocarditis, the risk scores we commonly use are not helpful in correctly assessing the patient's predicted risk^18^. Also in our study, some patients had a EuroSCORE II > 60/70%, which would have represented an absolute contraindication for intervention. In contrast, the surgical procedure in these patients was performed with good efficacy, indirectly suggesting the incomplete appropriateness of these scores in some cases of endocarditis.

In our opinion, a surgical and early approach should be adopted in these high-risk patients, as this strategy performs better than a “wait-and-see” or non-surgical approach, regardless of the predicted risk score. This opinion is shared by other colleagues who also addressed the issue of hospital costs, concluding that these patients should receive a rapid diagnosis and treatment in order to improve morbidity, mortality and reduce postoperative hospital costs^19^. The delay to surgery is not merely due to a “wait-and-see” approach but can also be related to diagnostic delays. In this regard, we fully agree with our colleagues who, following the current guidelines, by developing institutional protocols, have managed to reduce diagnostic times and, consequently, improve survival^20^. The take home message of our study therefore is that an early, completely and fast surgical approach might represent a valuable treatment in all high-risk patients, though larger studies are necessary to confirm our findings. These findings can be helpful to inform decision-making in heart team discussion.

## Data Availability

The datasets used and analysed during the current study are available from the corresponding author on reasonable request.
